# Supplementary material for: Chagas prevention and control in an endemic area from the Argentinian Gran Chaco Region: Data from 14 years of uninterrupted intervention
Source: PLoS Negl Trop Dis. 2023 Jun 14;17(6):e0011410. doi: 10.1371/journal.pntd.0011410 (PMC10266643; doi:10.1371/journal.pntd.0011410)
Supplement: S1 File — (DOCX) [file pntd.0011410.s001.docx]

| Settlement: ________________________________ | Date: ________________________________________ |
| --- | --- |
| Household ID: ______________________________ | \| Georeference: \|  \| Yes \|  \| No \| \| --- \| --- \| --- \| --- \| --- \| \| Photo: \|  \| Yes \|  \| No \| |

| **Profile of the Respondent – Responsible Adult*** | | | | | | |
| --- | --- | --- | --- | --- | --- | --- |
| Name |  |  | Sex | \|  \| Man \|  \| Woman \| \| --- \| --- \| --- \| --- \| | |  |
| Data of birth |  | Age | | |  | |
| Permanence in the settlement |  |  | Reads and writes? | \|  \|  \|  \|  \| \| --- \| --- \| --- \| --- \| \|  \| Yes \|  \| No \| | |  |
| \|  \| None \|  \| Kindergarten \|  \| Elementary \|  \| High school \| \| --- \| --- \| --- \| --- \| --- \| --- \| --- \| --- \| \|  \| Technical degree \|  \| University degree \|  \| **Other: \| \| \|   Schooling | | | | | |  |
| *A responsible adult is the person that provides economically for the home, if there is more than one individual that provides for the family, then the one that is “in charge of making the decisions” with respect to household expenses, is interviewed.  **In case that and individual only went to school until a certain grade or did not finish elementary or high school, indicate it in “other”. | | | | | | |
| **Inhabitants** | | | | | | |
|  | | | | | | |
| 1.- List all the individuals that live with the interviewee. Include the name and last name, relationship to the interviewee, schooling, date of birth and age of each one. With respect to schooling of children, write down the name of school they assist and grade they are in or until what grade they assisted.   \|  \| **Name and last name** \| **Relationship to respondent** \| **Schooling/Name of school*** \| **Reads and writes?** \| \| \| \| **Date of birth** \| **Age** \| \| --- \| --- \| --- \| --- \| --- \| --- \| --- \| --- \| --- \| --- \| \| **1** \|  \|  \|  \|  \| Yes \|  \| No \|  \|  \| \| **2** \|  \|  \|  \|  \| Yes \|  \| No \|  \|  \| \| **3** \|  \|  \|  \|  \| Yes \|  \| No \|  \|  \| \| **4** \|  \|  \|  \|  \| Yes \|  \| No \|  \|  \| \| **5** \|  \|  \|  \|  \| Yes \|  \| No \|  \|  \| \| **6** \|  \|  \|  \|  \| Yes \|  \| No \|  \|  \| \| **7** \|  \|  \|  \|  \| Yes \|  \| No \|  \|  \| \| **8** \|  \|  \|  \|  \| Yes \|  \| No \|  \|  \| \| **9** \|  \|  \|  \|  \| Yes \|  \| No \|  \|  \| \| **10** \|  \|  \|  \|  \| Yes \|  \| No \|  \|  \| | | | | | | |
| 2.- Have you or any member of your family ever had Chagas Disease? If affirmative pass to question 2a. | | | | | | |

| Yes | No |
| --- | --- |

| 2a.- Did you receive medical attention? If affirmative, pass to question 2b. |
| --- |

| Yes | No |
| --- | --- |

| 2b.- Did you receive any treatment? If affirmative, which treatment did you receive? __________________________ ________________________________________________________________________________________________ |
| --- |

|  | | | | |
| --- | --- | --- | --- | --- |
| **Socioeconomic variables and household characteristics** | | | | |
|  | | | | |
| 2.- Write down the approximate surface of the household : __________________________ (m^2^). | | | | |
| 3.- What is the main livelihood of the family? (More than one may be marked) | | | | |
| Animal raising | | Farming | | |
| informal laborer | | Salaried employment | | |
| Scrapyarding | | Craftsmanship | | |
| Public sector | | Retirement/Pension | | |
| Beneficiary of social plans | | Forestry | | |
| Tourism (hostelry/gastronomy) | | Other (please specify): _____________________ | | |
| 4.- The house is…? | | | | |
| Owned by the family | | Rented | | |
| Provided by employer | | Lent | | |
| Other (please specify): ___________________ | |  | | |
|  | | | | |
| 5.- What is the roof of the house made of? | | | | |
| Metal sheets | | Branches | | |
| Wooden boards | | Adobe | | |
| Palm leaves | | Other (please specify): _____________________ | | |
| 6.- What is the floor of your house made of? | | | | |
| Dirt | | Wood | | |
| Cement | | Bricks | | |
| Other (please specify): _____________________ | | | | |
| 7.- What are the walls made of? | | | | |
| Adobe | | Wood | | |
| Cement | | Bricks | | |
| Other (please specify): _____________________ | | | | |
| 8.- How many rooms do you use for sleeping? | | | | |
| 1 | | 4 | | |
| 2 | | 5 or more | | |
| 3 | | | | |
| 9.- Do you have a latrine or toilet? If affirmative, pass to question 9a. | | | | |
|  | | | | |
| Yes | | No | | |
| 9a.- What type of drainage does it have? | | | |  |
| Public sewer | | Sewer only | | |
| Septic tank and cesspool | | Cesspool only | | |
| Hole in the ground | | Other (please specify): _______________ | | |
| 10.- What do you use for cooking? (More than one may be marked) | | | | |
| Wood stove | | Stove with gas bottle | | |
| Stove with natural gas | | Clay oven | | |
| Other (please specify): _____________________ | | | | |
| 11.- Where do you obtain electricity from? (More than one may be marked) | | | | |
| Don´t have | | Electrical network | | |
| Generator | | Solar panel | | |
| Battery | | Other (please specify): _____________________ | | |
|  | | | | |
| 12.- Do you have a refrigerator or freezer to keep food? If affirmative, pass to question 12a. | | | | |
| Yes | | No | | |
| 12a.- What type of appliance do you have? | | | |  |
| Gas refrigerator | | Gas freezer | | |
| Electrical refrigerator | | Other (please specify): ________________ | | |
| 13.- What do you do with the garbage? (More than one may be marked) | | | | |
| Municipal service | | Open space | | |
| Burn it | | Burry it | | |
| Other (please specify): _____________________ | | | | |
|  | | | | |
| 14.- When you or one of your family members get sick, where to do you seek medical attention? | | | | |
| Health center | | Public hospital | | |
| Private medical office | | Health post | | |
| Other (please specify): ____________________ | |  | | |
| 15.- Do you or any member of your family have any of the following health coverages? | | | | |
| None | | Pre-paid medical care through work or healthcare plan | | |
| Voluntary pre-paid medical care | | Government health programs or social plans | | |
| Healthcare plan | | Other (please specify): _____________________ | | |
| **Questions related to water and hygiene** | | | | |
| 16.- Where do you get water for drinking? If the water used is not safe or potable, pass to question 16a. | | | | |
| Waterhole | | Water pump | | |
| Well | | Water tanker truck | | |
| Rain | | Bottled | | |
| Running water inside the house | | Running water outside the house | | |
| Water tank | | Other (please specify): ____________________ | | |
| 16a.- Do you treat the water to make it potable? If affirmative, what method do you use? | | | |  |
| None | | Boiling | | |
| Bleach | | Water filter | | |
| Other (please specify): _______________ | | | | |
| 17.- Where do you get water for cooking? | | | | |
| Waterhole | | Water pump | | |
| Well | | Water tanker truck | | |
| Rain | | Bottled | | |
| Running water inside de house | | Running water outside the house | | |
| Water tank | | Other (please specify): ____________________ | | |
| 18.- Where do you get water for bathing or washing hands? | | | | |
| Waterhole | | Water pump | | |
| Well | | Water tanker truck | | |
| Rain | | Bottled | | |
| Running water inside the house | | Running water outside the house | | |
| Water tank | | Other (please specify): _____________________ | | |
| 19.- Do you have a structure for washing your hands or dishes? If affirmative pass to questions 18a, b and c. | | | | |
| Yes | | No | | |
| 19a.- Do you have soap/detergent close to the struture? | | | | |
| Yes | | No | | |
| 19b.- Is the structure close to the latrine/bathroom? | | | | |
| Yes | | No | | |
| 19c.- Is the structure close to the place were you prepare food? | | | | |
| Yes | | No | | |
| 20.- Do you and your children usually wash your hand before eating? | | | | |
| Yes | | No | | |
| 21.- Do you or your children usually wash your hands after defecating? | | | | |
| Yes | | No | | |
| 22.- Do you or your children usually work barefoot? | | | | |
| Yes | | No | | |

|  | | | |
| --- | --- | --- | --- |
| **Peridomicile characteristics** | | | |
|  | | | |
| 23.- Do you have an animal pen? If affirmative pass to question 23a. | | | |
| Yes | | No | |
| 23a.- How is the animal pen made? | | | |
| With branches | | Wooden boards or trunks buried in the soil or held together by wire | |
| Other (please specify): _____________________ | | | |
| 24.- Do you have domestic or farm animals? If affirmative pass to question 24a. | | | |
| Yes | | No | |
| 24a.- Mark the animals that they have and in parenthesis add the approximate number of animals. | | | |
| Dogs | | Cats | |
| Gallinas | | Ducks | |
| Turkeys | | Sheep | |
| Goats | | Pigs | |
| Cows | | Horses | |
| Donkeys | | Rabbits | |
| Other (please specify): _____________________ | | | |

| 25.- Is your household usually sprayed or has it ever been sprayed? If affirmative, pass to questions 25a, b and c. |
| --- |

| Yes | | No | | |
| --- | --- | --- | --- | --- |
| 25a.- When was is last sprayed? | | | |  |
| 1 to 3 months ago | | 2 to 6 months ago | | |
| 1 year or more | | Other (please specify): _______________ | | |
| 25b.- Who performed the spraying? | | | |  |
| The Provincial Program | | Mundo Sano | | |
| The Municipality | | Other (please specify): _______________ | | |
| 25c.- What was the reason for spraying? | | | |  |
| Chagas | | Dengue | | |
| Cockroaches | | Other (please specify): _______________ | | |

|  |  |
| --- | --- |
|  |  |
