# Supplementary material for: Chagas prevention and control in an endemic area from the Argentinian Gran Chaco Region: Data from 14 years of uninterrupted intervention
Source: PLoS Negl Trop Dis. 2023 Jun 14;17(6):e0011410. doi: 10.1371/journal.pntd.0011410 (PMC10266643; doi:10.1371/journal.pntd.0011410)
Supplement: S3 File — (DOCX) [file pntd.0011410.s003.docx]

| **ENTOMOLOGICAL SURVEILLANCE AND CONTROL PROGRAM FOR CHAGAS DISEASE** | | | | | | | | | \|  \| \| --- \| |
| --- | --- | --- | --- | --- | --- | --- | --- | --- | --- | --- |
|  | | | | | | | | |  |
| **Record of chemical control of *Triatoma infestans* in households** | | | | | | | | |  |
|  | | | | | | | | |  |
| **Agent:** | | **Date: / /**  **Cycle of S&C:** | | | | | | |  |
| **Neighborhood/ Settlement:** | | **Block/Sector:** | | | | | | |  |
| **U N I T H O U S E H O L D D A T A** | | | | | | | | **C H E M I C A L T R E A T M E N T** | |
|  |  |  |  |  |  |  |  |  |  |
| **House ID** | **Name of responsible adult** | | **No. people** | **Ins.** | **Closed** | **Ret.** | **Dis.** | **No. of mono-doses** | **Observations** |
|  |  |  |  |  |  |  |  |  |  |
|  |  | |  |  |  |  |  |  |  |
|  |  | |  |  |  |  |  |  |  |
|  |  | |  |  |  |  |  |  |  |
|  |  | |  |  |  |  |  |  |  |
|  |  | |  |  |  |  |  |  |  |
|  | **T O T A L** | |  |  |  |  |  |  |  |
| **Observations:** | | | | | | | | | |
|  |  |  |  |  |  |  |  |  |  |
|  |  |  |  |  |  |  |  |  |  |

S&C: surveillance and control; ID: identification; No.: number; Ins.: inspected; Ret.: reticent; Dis.: dismantled.
