# Supplementary figures and images for: Chagas prevention and control in an endemic area from the Argentinian Gran Chaco Region: Data from 14 years of uninterrupted intervention
Source: PLoS Negl Trop Dis. 2023 Jun 14;17(6):e0011410. doi: 10.1371/journal.pntd.0011410 (PMC10266643; doi:10.1371/journal.pntd.0011410)

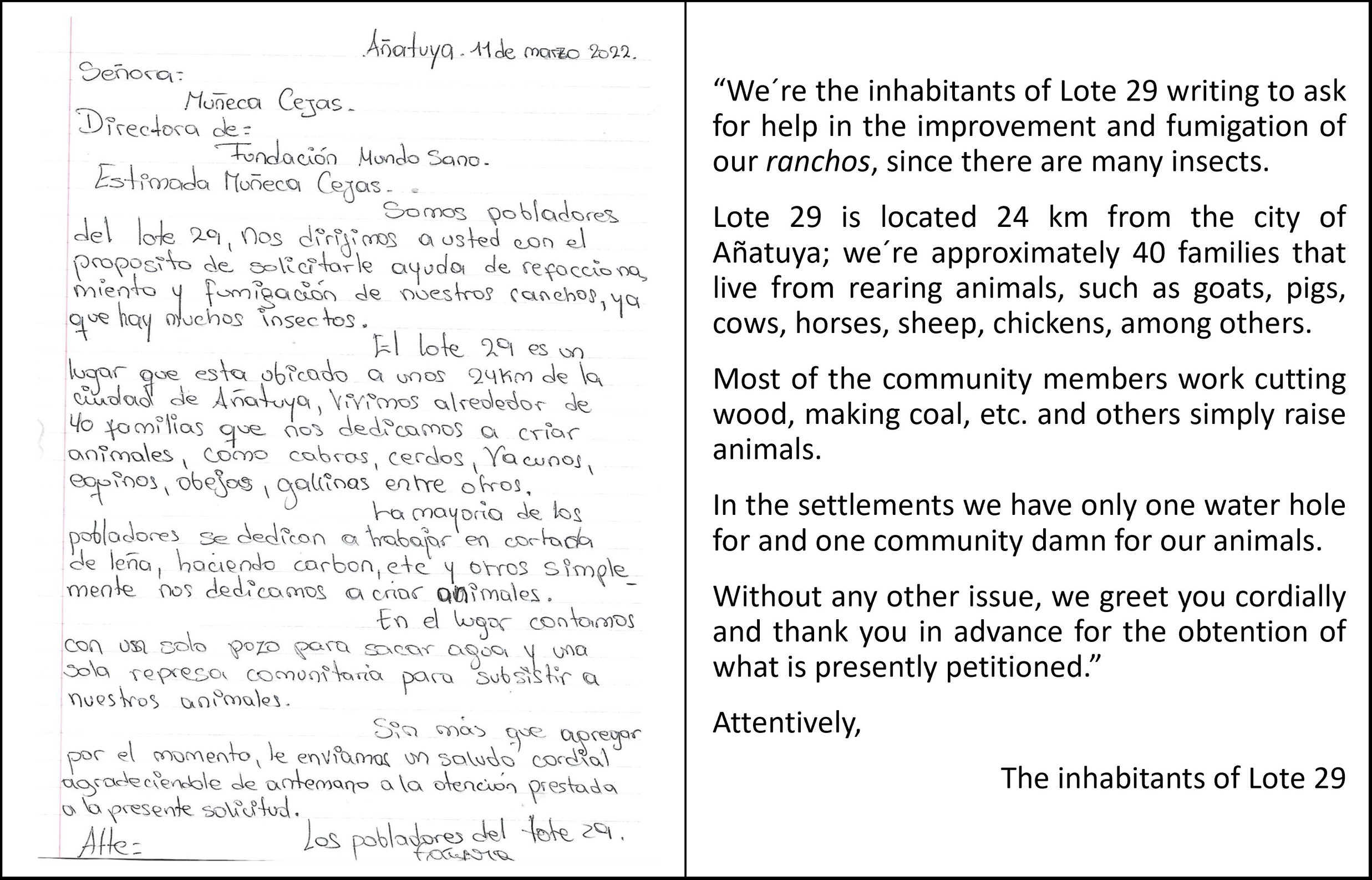

Supplement: S1 Fig — The panel on the right contains the transcription of the letter in English. (TIF) [file pntd.0011410.s004.tif]
