# Supplementary material for: Chagas prevention and control in an endemic area from the Argentinian Gran Chaco Region: Data from 14 years of uninterrupted intervention
Source: PLoS Negl Trop Dis. 2023 Jun 14;17(6):e0011410. doi: 10.1371/journal.pntd.0011410 (PMC10266643; doi:10.1371/journal.pntd.0011410)
Supplement: S1 Table — (DOCX) [file pntd.0011410.s005.docx]

**S1 Table.** Summary of yearly entomological indexes obtained during the different rounds of surveillance and control (S&C) implemented from 2005 to 2019 in the rural settlements from the Departments of General Taboada and Juan F. Ibarra, Santiago del Estero, Argentina.

| **Year** | **No. of settlements with S&C each year** | **No. of inspections** | **Overall infestation (%)** | **No. of houses with IDI (%)** | **No. of houses with PDI (%)** | **No. of houses with IDI & PDI (%)** |
| --- | --- | --- | --- | --- | --- | --- |
| 2005 | 1 | 39 | 18 (46.2) | 7 (17.9) | 6 (15.4) | 5 (12.8) |
| 2006 | 2 | 211 | 34 (16.1) | 22 (10.4) | 5 (2.4) | 7 (3.3) |
| 2007 | 3 | 202 | 27 (13.4) | 5 (2.5) | 16 (7.9) | 6 (3.0) |
| 2008 | 4 | 147 | 18 (12.2) | 3 (2.0) | 13 (8.8) | 2 (1.4) |
| 2009 | 3 | 92 | 39 (42.4) | 12 (13.0) | 16 (17.4) | 11 (12.0) |
| 2010 | 5 | 252 | 61 (24.2) | 20 (7.9) | 32 (12.7) | 9 (3.6) |
| 2011 | 6 | 247 | 35 (14.2) | 8 (3.2) | 25 (10.1) | 2 (0.8) |
| 2012 | 3 | 199 | 48 (24.1) | 7 (3.5) | 38 (19.1) | 3 (1.5) |
| 2013 | 7 | 280 | 76 (27.1) | 15 (5.4) | 51 (18.2) | 10 (3.6) |
| 2014 | 8 | 482 | 108 (22.4) | 9 (1.9) | 94 (19.5) | 5 (1.0) |
| 2015 | 9 | 413 | 52 (12.6) | 2 (0.5) | 50 (12.1) | 0 |
| 2016 | 10 | 546 | 68 (12.5) | 5 (0.9) | 62 (11.4) | 1 (0.2) |
| 2017 | 10 | 408 | 41 (10.0) | 3 (0.7) | 38 (9.3) | 0 |
| 2018 | 9 | 240 | 18 (7.5) | 1 (0.4) | 16 (6.7) | 1 (0.4) |
| 2019 | 12 | 435 | 22 (5.1) | 1 (0.2) | 20 (4.6) | 1 (0.2) |

*Infestation is calculated on the total number of receptive households each year. No.: number; IDI: intra-domiciliary infestation; PDI: peri-domiciliary infestation.
