# Supplementary material for: Chagas prevention and control in an endemic area from the Argentinian Gran Chaco Region: Data from 14 years of uninterrupted intervention
Source: PLoS Negl Trop Dis. 2023 Jun 14;17(6):e0011410. doi: 10.1371/journal.pntd.0011410 (PMC10266643; doi:10.1371/journal.pntd.0011410)
Supplement: S2 Table — (DOCX) [file pntd.0011410.s006.docx]

**S2 Table.** Summary of entomological indexes by settlement obtained during the different rounds of surveillance and control implemented from 2005 to 2019 as well as the number of insecticide applications, in the rural settlements from the Departments of General Taboada and Juan F. Ibarra, Santiago del Estero, Argentina.

| Settlement | No. S&C cycles (No. insecticide applications) | No. households_i_/ No. households_f_ | Neg_i_/Neg_f_ | Positive_i_/Positive_f_ | PDI_i_/PDI_f_ | IDI_i_/IDI_f_ | IDI+PDI_i_/IDI+PDI_f_ | McNemar or binomial exact test*  Y_i_/Y_f_ (p – value) |
| --- | --- | --- | --- | --- | --- | --- | --- | --- |
| El Desvío | 17 (12) | 39/55 | 19/51 | 20/4 | 9/0 | 8/4 | 3/0 | 2010 – 2019  (P < 0.01) |
| Miel de Palo | 18 (13) | 68/91 | 41/83 | 27/8 | 20/1 | 0/7 | 7/0 |  |
| La Salamanca | 21 (14) | 33/39 | 32/39 | 1/0 | 1/0 | 0/0 | 0/0 |  |
| Lote 28 | 14 (7) | 2/9 | 0/9 | 2/0 | 2/0 | 0/0 | 0/0 |  |
| Pozo Herrera | 12 (8) | 32/37 | 21/37 | 11/0 | 0/0 | 11/0 | 0/0 |  |
| Lote 46 | 9 (8) | 8/14 | 4/13 | 4/1 | 1/0 | 2/1 | 1/0 | 2011 – 2019 (P = 0.25) |
| Lote 47 | 7 (6) | 47/46 | 23/44 | 24/2 | 8/0 | 7/2 | 9/0 | 2013 – 2019 (P < 0.01) |
| Lote 27 | 8 (7) | 20/24 | 8/21 | 12/3 | 1/0 | 9/3 | 2/0 | 2014 – 2019 (P < 0.05) |
| Lote 58 | 5 (5) | 43/54 | 34/53 | 9/1 | 0/0 | 9/1 | 0/0 | 2015 – 2019 (P < 0.05) |
| Lote 59 | 4 (4) | 5/9 | 1/6 | 4/3 | 0/0 | 4/3 | 0/0 | 2016 – 2019 (P = 0.25) |
| Los Pocitos | 4 (4) | 34/32 | 23/32 | 11/0 | 1/0 | 10/0 | 0/0 | 2017 – 2019 (P < 0.01) |
| Lote 28 Grande | 2 (2) | 27/25 | 22/25 | 5/0 | 1/0 | 3/0 | 1/0 | 2018 – 2019 (P = 0.25) |
| Malacara | 1 (1) | 38/NA | 37/NA | 1/NA | 0/NA | 1/NA | 0/NA | NA |

No.: number; S&C: surveillance and control; i: initial; f: final; Neg: negative; IDI: intra-domiciliary infestation; PDI: peri-domiciliary infestation; Y: year; NA: non-applicable. *Statistical comparison of paired households was performed using overall positivity regardless of place of occurrence (intra or peridomestic).
