# Supplementary material for: Chagas prevention and control in an endemic area from the Argentinian Gran Chaco Region: Data from 14 years of uninterrupted intervention
Source: PLoS Negl Trop Dis. 2023 Jun 14;17(6):e0011410. doi: 10.1371/journal.pntd.0011410 (PMC10266643; doi:10.1371/journal.pntd.0011410)
Supplement: Attachment — Submitted filename: Response to reviewers.docx [file pntd.0011410.s007.docx]

Response to reviewers

We would first like to thank the time taken by the reviewers and editorial team in reading and reviewing the manuscript. Your valuable suggestions and commentaries have helped to improve the work. We greatly appreciate it.

Please find the point-by-point responses below.

This paper describes the effect of a long-term evolving intervention in the Argentine Chaco that decreased triatomine infestation over time. In addition to the comments mentioned by the reviewers I have a few suggestions:
- It would be helpful to define "ecohealth" in the introduction.

This was moved from the method section to the introduction.

- Table 2 needs some explanation about why there are more houses improved than currently exist in some sites.

The last column in Table 2 refers to the cumulative number of houses improved; therefore, it adds the houses from the previous settlement mentioned in each line, in the order they were incorporated in the program. In total, there are currently 527 houses in the 13 settlements, and 399 (around 75% of these were improved). This was clarified in the Table 2 description to avoid confusion.

- If available, it would be interesting to report the prevalence of Chagas disease in specifically in children (esp. <5 years old) in these areas over the course of the intervention, as this more accurately reflects ongoing transmission.

Unfortunately, the data available publicly through the national notification system, does not provide private information such as age. Data from a project conducted in rural settlements from General Taboada in 2003 showed and overall prevalence of *T. cruzi* of 5.8% in children from 6 months to 14 years, with a specific prevalence of 0 in children 6 months to 2 years and 2.6% in children from 3 to 5 years. But the total number of tested children was 489. The highest prevalence, 5.7%, was observed in children 6 to 14 years old. This data was added in the discussion. Data from more recent serology performed by the local authorities with Mundo Sano was also added and the data from the national system was removed.

- The discussion could benefit from addressing sustainability of the intervention and its applicability in other endemic places (Bolivia, etc).

This has been added and addressed in the discussion as suggested.

**Editorial and Data Presentation Modifications?**

Use this section for editorial suggestions as well as relatively minor modifications of existing data that would enhance clarity. If the only modifications needed are minor and/or editorial, you may wish to recommend “Minor Revision” or “Accept”.

Reviewer #1: (No Response)

Reviewer #2: A thorough revision of the English is strongly suggested. Spanglish seems to occur (for ex. Line 214 : deposit instead of storerooms).

This was revised though out the manuscript as suggested.

Minor comments
Please give the url to access reference 19. This reader could not find it on the web

This URL was added as suggested.

Ref 20, 45 and 43: I suggest including the corresponding urls to help accessing these references.

These were added as suggested.

L227: There is a typo: “but if not adult or nymph bugs are observed,”

This typo was corrected, thank you.

Suppl File 3: Please explain the abbreviations included in this record sheet.

Abbreviations were included as suggested.

Reviewer #3: The manuscript can be enhanced by an English style revision.

Language was revised as suggested.

--------------------

**Summary and General Comments**

Use this section to provide overall comments, discuss strengths/weaknesses of the study, novelty, significance, general execution and scholarship. You may also include additional comments for the author, including concerns about dual publication, research ethics, or publication ethics. If requesting major revision, please articulate the new experiments that are needed.

Reviewer #1: Dear authors,

Thank you for providing me with the opportunity to review this manuscript. This study presents data from the 14-year field engagement, which are highly worth being published. However, the current version of the manuscript reads more like a project report, rather than a scientific research paper. I encourage the authors to consider the following points:

A) What is your research question(s)?
B) Distinguish clearly between the observers (“the study”) and the observed (“the program”). It may be hard to separate them in this kind of work because the authors have been implementing and studying the program at the same time. However, at least conceptually, these two endeavors need to be distinguished.
C) Try to make some inferences, whether descriptive or causal. By inferences, I mean that we say something about unobserved facts using the facts we have observed.

Among many possible ways to improve this manuscript, the authors may choose:

1) Reframe the study as an assessment of the echohealth intervention. The study will assess the impact of the 14-year intervention on entomological indicators.
2) First, describe the contents and processes of interventions.
3) Second, describes the longitudinal trends in entomological indicators.
4) Then, discuss whether the observed changes (or sustained reductions) in entomological indicators can be attributable to the intervention. To what extent is the echohealth intervention effective in reducing vector populations?
5) If you have data, it may be helpful to compare the entomological index between the settlements with and without the echohealth intervention.

Please note that the authors do not have to exactly follow these guidelines from 1) to 5), but I hope they can give the authors some ideas to improve the manuscript.

Thank you for helping us organize the writing in a more comprehensive manner, indeed, after 14 years of intervention, there is a lot of information and data and there are many situations that have arisen throughout the years. We have tried to focus the paper as suggested, into an assessment of in the intervention itself, taking into consideration the work that allowed for FMS to be present and active through these 14 years, as well as the impact these actions had on the entomological indexes of the different settlements. Unfortunately, and this is mentioned as a limitation of the intervention, we do not have information on settlements without the intervention, although we do have the baseline entomological indexes versus the final ones (2019).

Reviewer #2: The title of this article focus on data on the ongoing project but the data is not available. I suggest changing the title and also including the relevant data as a supplementary material.

The title refers to the 14 years of intervention from 2005 to 2019, it does not include settlements that were added after 2019. Although all the settlements are under surveillance and control until today, we had to make a cut somewhere to be able to analyze the data in a yearly manner. Also, during the pandemic, the activities had to be interrupted during different time lapses and therefore we decided to report all the data before the year 2020 for uniformity. Relevant data was added as supplementary material, as suggested.

The authors state “Therefore, the objective of the current study is to show the evolution of the program” as this is a research article, the authors need to clarify which is the research question. For this reader describing the evolution of the program is not worth publishing in Plos NTD, especially if no figures on the costs invested in the program are included or no relation between the program described and the expected outcomes are inquired. No figures are included on the amount of effort in control actions, for instance insecticide spraying (number of houses sprayed, number of houses re-sprayed per community, etc).

The objective of the study is to describe the different component of the ecohealth approach used, which is ongoing and maintained since 2005, therefore part of that includes reporting the evolution of the program and the impact it had on the entomological indexes. We believe this is useful for the readers of PLoS NTD, given it deals with an NTD, Chagas Disease, which is highly prevalent and remains a problem to this day, despite efforts from national programs dedicated to its elimination. In this manuscript, the description of the approach, with the availability of standard operating procedures, implementation in various settlements with local authorities and community participation, show that reduction in infestation indexes were achieved and this has impacted on a reduction in infection rates in the local population (this data was added as suggested by another reviewer).

Abstract
Lines 26-28: The authors state “Argentina´s ChD program was originally vertical and centralized; later, it was partially and unsuccessfully transferred to the provinces” Is this a generalized fact? How can the authors explain the fact that there are endemic provinces were success has been achieved (i. e.: measured as certification of vector-borne domestic T. cruzi interruption?

This statement was corrected given that in some provinces of Argentina, certification was achieved.

Lines 40-41: “Intra and peri-infestations” refer to intra-domiciliary and peridomestic infestation? I suggest the authors spell out these words for sake of clarity.

This was corrected.

Author summary
L49-52: Please correct Triatoma is only the main vector in The Gran Chaco region and Arequipa. The authors need to narrow down their affirmations.

This was modified as suggested.

L56-58: Please revise the writing of this sentence “the most cases of ChD” sounds incorrect.

This mas modifies to greatest number of ChD cases.

Introduction

L 99-101. “Despite these public health measures, the national control of ChD still eludes us due to inconsistency in the application of these measures, a problem that transcends our borders “
It is not clear to this reader what is eluded. (“us” seems to be a mistake in this sentence).

This sentence has been modified to “the national control of ChD has yet to be achieved due to inconsistency in the application of these measures…”

L115-116 “Santiago del Estero is located within the Gran Chaco Region, which is historically one of the regions with the most cases of vector transmitted ChD [22, 23].” Please revise this expression.

This expression was revised to “which is considered a hot spot for NTD´s, including ChD.”

L122 The term “ecotope” to refer to the monte forest seems inaccurate.

This was modified to “ecoregion”.

L133 and 134: Ref 33 for these lines is incorrect. I suggest citing Gürtler et al., 2009. PNAS 104 (41) 16194-16199. <https://doi.org/10.1073/pnas.0700863104>

The reference was modified as suggested. The article the reviewer suggested citing was already listed as reference number 7.

M&M
I suggest using Past Tense

The section was modified using the past tense as suggested.

A timeline indicating when each of the activities took place and when each community entered the entomological program and the housing improvement program would be very helpful.

A timeline was included as Figure 2, following the reviewer’s suggestion.

L 342-343. “The interior of the houses, roof improvement and waterproofing have remained the same to this day” What is the meaning of this phrase? These improvements were performed in 2005 and remained unchanged from that onwards or the type of improvement has not changed along the program? Please clarify.

This was clarified in the text. These improvements remained the same since 2005 until today but other improvements were modified over time and detailed in the manuscript.

Results
L366-368. Indeed in the Supl material Fig 4 included as an example, the community is asking for insecticide spraying (not entering to the program as stated in L366-368). Please rewrite these sentences for sake of accuracy.

The program as offered in the rural areas, with PFIP funding, started with surveillance and control and continued with household improvement, all of these components, including insecticide spraying are part of the program. Given the longevity of the program, not all the letters were digitalized, and some got lost along the way. Nonetheless, the letter was changed by one from a settlement that sent a letter last year and they specifically ask for both entomological control and home improvements.

Table 2 should be unified with Table 1

Initially we had only one table, but unification of them generated more confusion given that surveillance and control on one side and house improvements on the other, are performed at different times when several settlements are under program at the same time. This is why, for clarification, we have divided the information in two tables.

L 389-392. My main suggestion regarding results is to re-analyze and rewrite this paragraph and the following ones that refer to the entomological results. As they are right now, results from baseline infestations are joined with results from communities that may be in the surveillance phase just because field work took place at the same year. Thus, I suggest expressing results in reference to the number of cycles and not to the year when they took place as it is in Figure 3 but for all the communities involved.

Table 1 summarizes the progress of the program and the number of surveillance and control cycles completed in each settlement. Since this program has been implemented continuously since 2005, and has comprised 13 settlements up to 2019, analyzing one by one would make the manuscript too long, therefore we decided to show the data of the program in its entirety. We have now included a timeline to make interpretation easier (as suggested by the reviewer). We do agree that the infestation indexes vary depending on the number of S&C cycles and therefore we have added some information from specific settlements to show this in Figure 3, the dates reflected in the 4 settlements shown in this figure are in fact the different cycles: 17 for El Desvío (Fig. 3A), 18 cycles for Miel de Palo (Fig. 3B), 21 cycles for La Salamanca (Fig. 3C and 12 cycles for Pozo Herrera (Fig. 3D); this was clarified in the text and in the figure legend. Nonetheless, as stated in the discussion, other factors influence on the fluctuation in the entomological indexes, especially the peri-domestic infestation index were passive transfer of triatomines from one place to another or the absence/presence of animals also play a role.

In order to provide more data, we have added Supplementary file 6: Table 2 with the variation in entomological indexes by settlement according to the number of S&C rounds with statistical data.

Is there an explanation for the abrupt increase in infestation levels observed in La Salamanca (Figure 3C)? It seems to occur in january 2010, i.e.: after massive insecticide spraying of all the community and after the housing improvement was launched? Who looked for triatomines in these houses? Has the same team performed triatomines searches in 2007 -2008 and 2010 in La Salamanca?

The team performing surveillance and control of the settlements is a stable and permanent team from Mundo Sano, given the organization has a permanent office in the city of Añatuya since 2002. There has been some changes in personnel over the years, not many, but regardless, there is an SOP specific for this task and new hires are trained specifically.

Each of the settlements, have gone through their different demographic and constructive changes, as the years have passed and concretely in La Salamanca, there was a process of re-localization of families during 2009-2010, with the construction of new houses, therefore deforestation took place to open an area for construction of the new houses and there was dismantling of the old ones, this had an influence on the presence of kissing bugs, increasing their number. In this settlement, the construction of a club with a field also took place with deforestation, this is why we can observe a large fluctuation in the entomological indexes from 2010 to 2014. This was added in the discussion.

Please include the number of houses sprayed with insecticides per year per community.

This was included as a new Supplementary file: Table 3.

Please include de number of houses sprayed in figures 2 and 3.

We believe this will make the figures too busy so we have added a supplementary file with this data.

L417-420 repeat information from M&M. I suggest deleting them.

These were deleted as suggested.

Discussion
The impact of the program on T. cruzi transmission is not addressed in this manuscript.

This was added in the discussion as suggested.

Conclusions:
L577-8:“The program described within has shown that the ecohealth approach is effective for long term surveillance and control of ChD.” At what cost? If this program is to replicated elsewhere, which is the input needed?

We have modified the discussion as to take this into consideration.

Reviewer #3: Comments to the authors

The work presented is very interesting and deals with a long term problem of the region. This integrated and community based management program is promising and already showing good results. With respect to the manuscript I encourage authors to look for some shortening of the text, which can be achieved by reorganizing the information and avoiding redundant phrases. It would be interesting to show statistical information regarding the comparison of peri and intra domestic infestation. The discussion section dedicates some lines to covering results. This is unnecessary and the discussion would be enriched by comparing the results of this program with other programs that included, or not, house improvements. This could be done by including more details on cited work.

Thank you for your input. Your suggestions have been taken into consideration and the text was modified for clarity, it was shortened in some areas, statistical analysis was added and the discussion was modified.

Specific comments
Line 77-80 This seems unclear, please rephrase. I suggest in line 79 replacing “and currently….into” for “corresponding to”

This sentence was modified for clarity.

Line 93 Mentioning “Almost 20 years later“ sounds awkward in this context, I suggest starting the line directly with “In 1980”

This sentence was modified as suggested.

Lines 112-114. This is mentioned later in lines 147-150. Please organize the text for one mention only.

The text in these two areas of the introduction was organized to avoid repetition, as suggested.

Lines 119-123 “More specifically…..Lived”. This looks more appropriate for the materials and methods section.

This was left in the introduction because it is tied to the next paragraph (lines 127-133).

Lines 144-146 “With…..evolved” looks redundant. I suggest erasing this sentence and modifying the following, eg “By 2005 our program, that had started in urban areas, was expanded to….·

This sentence was modified as suggested.

Line 148 Replace “the component…. was added” by “This was done” or similar. In general there are several mentions to “the improvement of households” so I encourage authors to look for ways for rephrasing the term.

The text was modified taking the reviewer´s comment into consideration.

Line 150 Remove “individuals”

Removed as suggested.

Line 173 -174 Is this total number of households? If so, please add “total”. Remove “in the settlement”. Please provide some details on the meaning of “previous relation with FMS” and “organization of the community”. The rest of the paragraph corresponds to Type of experimental design so the authors may rephrase the title of the section.

These comments were taken into consideration and the text was modified as suggested.

Line 206-207 The request for consent is mentioned in line 191. I suggest including that consent was requested in each visit in line 191, and erasing that in lines 206-207.

This was erased as suggested.

Line 219 Rephrase “If at least one T. infestans individual is found the house is considered positive, and details on the specimen /s found, such as place of discovery (…), life stage (…) and …. is recorded”.

This sentence was rephrased as suggested.

Then the line 223-224 “A house…. both” can be removed. If presence of other triatomine species determined positivity please mention.

This entire paragraph was rephrased.

Lines 236-238 Because the collected information was mentioned earlier, this sentence could be shortened to saying that all the information gathered was included in the platform, and moved to Statistical analysis section.

This mas modified as suggested.

Line 238-240 This sentence may fit better at the beginning of the section, when the overall process of the program is presented. Line 253 In a) you could say “positive houses”

This was changed as suggested.

Lines 263-265 Is this the same information as in lines 250-251? If so, please remove one mention, otherwise please rephrase for clarity

This mas modified as suggested.

Lines 265-271 Looks better suited for Study Area section.

The section of the paragraph was changed to the house improvement section.

Line 291 I suggest replacing “In order….. household” by “Secondly”

This sentence was modified.

Line 295 I suggest replacing “ according….. defining” by “as”

This sentence was modified.

Line 303 While some habits and building structures can facilitate the settlement of triatomine populations I would be cautious of saying they “lead” to infestation. Possibly a different word would better represent reality.

This was changed to “may favor the presence of…”

Statistical analysis: In this section you could include a line mentioning which information was recorded and saved.

This was added as suggested.

Lines 360-361 Can you please be more specific on what was compared?

This was clarified.

Lines 424-429 This description could be improved by making statistical comparisons. Are the insects statistically more frequent in peridomestic areas? Is some peridomestic area statistically more infested? Are the insects statistically more frequent in bedrooms than is other areas of the intra domicile?

Statistical comparisons were made and added to the text.

Lines 451-459 Most of this information was mentioned earlier. Please revise and delete redundant information.

This was revised to avoid redundancy.

Line 468 Delete “these were taken…… herein”

This was deleted as suggested.

Line 471-474 Starting at “different…” should be moved to the next paragraph. For example at line 483 delete “unlike this type of programs” and insert the previous sentence eg as this “Because different studies highlight …households, “and” tackling…..insufficient”, the experience described herein……

This was modified as suggested.

Line 502 Authors may be able to find details on infestation rates of other areas that were not part of this study, either at governmental Chagas disease programs or per request to other authors working in the topic (eg the group of Ricardo Gürtler). Showing some type of comparison with areas that only received insecticide treatment (and where no resistance to pyrethroids has been described) would be very interesting.

Unfortunately, this data is not available. Gürtler´s group has worked in other areas of Santiago del Estero, located around 200 km away from Añatuya, therefore, we don´t think the data is comparable.

Lines 515-524 This part of the paragraphs is rather redundant with previous information appearing in the text. This could be reduced to one sentence that opens the following discussion on the ecohealth approach.

This was reduced as suggested.

Lines 531-533 This is important, as sometimes the effectivity of insecticide spraying is decreased by inconsistent periods of application, insecticide resistance and the impossibility to access every house in a given area, among other issues. There is evidence that the residual effect of insecticides is different depending on the construction material (eg Germano et al 2014 “Fenitrothion….”, Gürtler et al 2004 “Effectiveness…”, Rojas de Arias et al 2004 “Pyrethroid…” and others) so house improvements may enhance the result of spraying by providing a different wall plastering.

We agree and have added the references that were not previously mentioned.

Lines 549-554 The failure of a program for eradicating ranchos was discussed earlier by lines 475 and on. Please reorganize the text for only one mention of this.

These lines were removed as suggested.

Lines 564-574 This paragraph is rather redundant with previous ones. Possibly it could be resumed in one or two sentences mentioning that further studies are required to determine the role of the settlement characteristics and of the natural areas in the differences of infestation between areas.
Supplementary material. Letter from Lote 27. The letter requests insecticide spraying. Did the community request other involvement in the program? Did any other community request participation?

The letter is an example, FMS has received letters from different communities, but they have not all been digitalized, and some were lost along the way. We have added another more recent letter (2022) from the new lot, Lote 29, which I will be using to substitute the one from Lote 27. Given that FMS implements both S&C and home improvement as one project, some communities did not specify that in their letter. This is because the program starts with S&C and then continues with improvement, homes are not usually improved unless S&C is previously performed.
