# Supplementary material for: Chagas prevention and control in an endemic area from the Argentinian Gran Chaco Region: Data from 14 years of uninterrupted intervention
Source: PLoS Negl Trop Dis. 2023 Jun 14;17(6):e0011410. doi: 10.1371/journal.pntd.0011410 (PMC10266643; doi:10.1371/journal.pntd.0011410)
Supplement: Attachment — Submitted filename: Response to reviewers.docx [file pntd.0011410.s008.docx]

Response to reviewers

Thank you for your time with the comments and suggestions, we hope that they have all been properly addressed.

**Summary and General Comments**

Use this section to provide overall comments, discuss strengths/weaknesses of the study, novelty, significance, general execution and scholarship. You may also include additional comments for the author, including concerns about dual publication, research ethics, or publication ethics. If requesting major revision, please articulate the new experiments that are needed.

Reviewer #1: Dear authors,

Thank you for your efforts in revising the manuscript. As a general comment, I strongly recommend the authors review the manuscript carefully and thoroughly to avoid rejection in the next review round. I provide the list of specific comments below, with some points that must have been corrected by the authors before the authors asked reviewers to work. More importantly, please do not consider this list exhaustive: responding to these comments only may not be sufficient to improve the quality of writing to the acceptable level.

Line 99: What does “national control of ChD” mean? When can it be “achieved”?

This has been modified as suggested, the control of ChD, according to the milesontes set by the new roadmap for NTDs (WHO), has yet to be achived.

Line 100: Who are “our”?

This sentence has been modified to make it clear that the situation seen in Argentina is the same as in other countries.

Line 104-5: It seems that this paper describes more than the S&C program, according to the Method section.

This was clarified as suggested.

Line 129: “households” should be “houses,” if you are talking about physical structures.

This was changed from households to houses as suggested.

Line 131: There is a logical leap. What connects settlements and serological studies?

This paragraph has been modified to clarify that historical data report the presence of settlers and triatomine bugs in the area, but published serological and infection data in these settlers is scarce, although we were able to get some information on serology in humans and infection in bugs from a few published studies, showing there has been a decrease.

Line 132-3: two? Two departments in the Province of Santiago del Estero?

This was modified, since in fact, the studies are from more than two department. We just wanted to show that there are very few localities with data.

Line 145: Check whether you talk about the S&C program only here.

Yes, we have added the word original since in the urban area, where the program started, improvement in the hoses were not included.

Line 163-4: I don’t see any data or relevant arguments about access to diagnosis and treatment in this paper. This mention of diagnosis and treatment should probably be removed.

This was modified to reflect that given the sustained control achieved, these populations can now access diagnosis and treatment, with minimal risk of re-infection.

Table 1: “No. of settlements included” should be “Cumulative number of settlements included”

This was modified as suggested.

Table 1: “Current No. of completed S&C cycles” --- when is “current”? As of when?

This is the number of completed cycles as of 2019, this was clarified in the table as a footnote.

Table 1: “Current No. of completed S&C cycles” --- it’s easier to read the table if “cycle” is explained in the table’s footnote (its definition appears far in Line 240).

This was added as a footnote as suggested.

Line 370-79: These sentences seem to merely describe Table 1, which is not necessary.

These were removed as suggested.

Line 391: Please consider the numbering of supplementary files and tables. I thought you were referring here to Table 2 in the main text.

This table is numbered as Supplementary file 6.

Line 420: What are “findings”?

This refers to the places where the bugs were found and was clarified in the text as suggested.

Line 449: The paper describes more than the S&C program.

This was modified to reflect that a S&C program with an ecohealth approach is being described.

Line 461-71: What is the point of this paragraph?

This paragraph details the work that has been performed with respect the importance of the characteristics of the houses that favor the presence of triatomine bugs to show that this is not something new, these previous experiences served as the basis for the experience described in the manuscript.

Line 472-89: What is the point of this paragraph?

Likewise, this paragraph describes the work that the provincial government has been performing with respect to housing and specifies the failure of the eradication of the rancho given that the inhabitants favor their traditional house.

Line 490: What is “this virtuous association”?

This was modified to reflect that the use of an ecohealth approach in a public-private association, has allowed for the program to continue to this day.

Line 519: What does “This” refer to?

This was clarified in the text.

Line 521: “were”?

This sentenced was modified.

Line 521-22: Which “individual and social determinants” were considered indeed?

As stated, a few lines below, providing improvements and listening to needs that went beyond the specific problem of ChD were considered. This refers to the water and basic sanitation improvements that were made in the houses, not only characteristics that are related to the presence of bugs in the house. Also, the improvement of the rancho and not eradication of the structure itself.

Line 529: It’s not clear why the authors consider insecticide as a probable key driver to lowering vector infestation. Please make your arguments clearer.

This was modified as suggested, clarifying that only the improvement of the household is not enough to drive down the population of triatomine bugs in the peridomicile in rural areas where the sylvatic cycle is ongoing, and you have animals in that serve as bloodmeal for the kissing bugs.

Line 529-32: So, are you arguing here that insecticide alone is OK to control vector populations or are you insisting that insecticide should be combined with other measures? Make your claim clearer.

This has been modified to argue that both are necessary, improvement of houses and insecticide spraying.

Line 533-549: These two paragraphs contain lots of serological data. Where do these data come from? If they come from the already published materials, please put citations. If they are original data of this paper, the authors should describe how these data were collected and analyzed in the Method section and report findings in the Result section.

These are findings from serological studies performed by the municipality and reported to FMS, as stated in the discussion. These were included by petition of the reviewer, but they are not the object of the program presented. We have removed them.

Line 550: “knowledge” about what? What are “technological tools”?

These were specified in the text.

Line 554: “access to technology” --- which technology are you talking about?

This was specified in the text.

Line 561-9: What is the point of this paragraph? The first sentence is too long to figure out the authors’ main claim.

This paragraphs and the following were modified and united to drive the point of the importance of recording, documenting, and having SOPs to be able to replicate the program but also, the adaptability to local customs and situations.

Line 570-81: What is the point of this paragraph? Are you trying to explain differences observed among settlements?

This paragraph was shortened and united with the previous one and explains that there are still certain environmental and land usage characteristics that need to be explored to fully explain the transmission patterns that we are seeing in this area.

Discussion Section: As the authors pointed out in Introduction, many Latin American countries have experienced decentralization of vector control programs and moved towards PHC-integrated or community-based vector surveillance systems (e.g., see Hashimoto et al. BMC Health Serv Res. 2015; Yoshioka et al. Infect Dis Pov. 2017). After 14 years of implementing the S&C program in Argentina, do the authors insist that the Government of Argentina should hire field agents to maintain this kind of S&C program? Does it economically feasible and sustainable? If not, what could be an alternative?

This was modified and now clearly mentioned in the conclusions.

Line 583-4: What do you mean by “the ecohealth approach is effective for long term [sic] S&C of ChD”? This sentence is confusing because S&C seems to be a component of the “ecohealth approach,” according to Lines 180-4. If so, S&C is not an outcome of the “ecohealth approach.”

This was modified as suggested.

Line 586: Where is the evidence for “< than [sic] 1% seroprevalence”? Data are not properly presented for this claim.

This was removed.

Line 584-587: How do all of the evidence guide control programs? What are the authors’ specific recommendations?

This was specified as suggested.

Line 593-4: “has enabled the population to get access to diagnosis and etiological treatment of ChD” --- this claim is not supported by data or relevant arguments in the paper.

This was changed to reflect it as a possibility in the future.

Reviewer #2: All my previous comments have been addressed
Minor comment:
The url of reference N 64 seems not to work. Please verify

This URL was verified and is working properly.
